# The Ameliorative Effect of *Zingiber officinale* in Diabetic Nephropathy

**DOI:** 10.5812/ircmj.11324

**Published:** 2014-05-05

**Authors:** Mahmoud Rafieian-Kopaei, Hamid Nasri

**Affiliations:** 1 Medical Plants Research Center, Shahrekord University of Medical Sciences, Shahrekord, IR Iran; 2 Department of Nephrology, Division of Nephropathology, Isfahan University of Medical Sciences, Isfahan, IR Iran

**Keywords:** Diabetic Nephropathies, Ginger, Renoprotection

Dear Editor,

Diabetic nephropathy is a progressive kidney disease and is characterized by diffuse glomerulosclerosis and nephrotic syndrome. Diabetic nephropathy is due to angiopathy of capillaries in the kidney glomeruli and is a prime indication for dialysis. It might be seen in patients with diabetes mellitus (DM) after about five years in type 1 diabetes and affects 25% to 35% of patients under the age of 30 years. Diabetic nephropathy is a progressive complication of DM that may lead to death two or three years after the initial injury. It is the end-stage kidney disease and the most common cause of chronic kidney failure. The risk of diabetes nephropathy is higher if blood glucose level and blood pressure are poorly controlled. Furthermore, people with high blood cholesterol level have much higher risk than those with normal cholesterol level (1-4). Nowadays attention has been drawn toward the possible kidney protective properties of *Zingiber officinale* (ginger) in these patients. To figure out the ameliorative effect of ginger extract against tubular damage induced by gentamicin, we conducted an experimental study in 60 male Wistar rats (6 groups of 10 rats). We treated them as follows: group I, vehicle; group II, ginger for three days and then gentamicin for seven days; group III, ginger orally for three days and then ginger plus gentamicin for seven days; group IV, gentamicin for seven days; group V, gentamicin for ten days; and group VI, gentamicin for seven days and then ginger orally for ten days. At the end of the study, kidneys were removed for histological evaluation. In this study, we observed that ginger could prevent degeneration of the renal cells and reduce the severity of tubular damage caused by gentamicin. We concluded that ginger was effective as a prophylactic agent for renal tubular cells against the acts of injurious substances like gentamicin (5). In the study conducted by Tzeng et al. the ameliorative effects of ginger on renal damage in diabetic rats was investigated. In their study, diabetic rats were treated orally with ginger or metformin for eight weeks. They found that ginger displayed similar characteristics to those of metformin in reducing hyperglycemia and renal dysfunction in diabetic rats. The histological examinations revealed amelioration of diabetes-induced glomerular pathological changes following the treatment with ginger. Furthermore, the protein expressions of renal nephrin and podocin in diabetic rats were significantly increased following the treatment with ginger. They suggested that the renoprotective effects of ginger might be similar to the action of metformin in the prevention of AMP-activated protein kinase protein phosphorylation (6). Metformin has been widely used in the patients with DM, especially in type 2 DM (7-10). Recently, the possible renoprotective role of metformin was noticed (7-10). In the study of Morales et al. ameliorative properties of metformin against gentamicin-induced renal tubular damage were shown (11). To find the potential efficacy of metformin in protecting kidney against gentamicin-induced acute kidney injury and to test whether delay treatment with metformin in acute kidney injury exerted similar efficacy on gentamicin-renal injury in rats, we conducted a study on Wistar rats. We found that metformin was able to prevent and ameliorate gentamicin-induced acute renal injury. Hence, it might have renoprotective efficacy (12). More recently, we observed the efficacy of coadministration of garlic extract and metformin in prevention of gentamicin–induced renal damage in 70 male Wistar rats (13-15). The result of these studies showed that metformin and ginger juice had ameliorative effects against gentamicin-induced nephrotoxicity. The protective effect of metformin on diabetic nephropathy was also recently published by Kari et al. (16). The incidence of DM and nephropathy of diabetes have risen rapidly (17-19). Therefore, the combination of metformin and ginger extract might be more effective to control of DM and might have additive protective efficacy on diabetic nephropathy. To better figure out the protective effect of ginger on kidney, particularly in combination with metformin, more researches are needed.

## References

[1] Nasri H (2013). On the occasion of the world diabetes day 2013; diabetes education and prevention; a nephrology point of view.. J Renal Inj Prev..

[2] Rahimi Z (2012). ACE insertion/deletion (I/D) polymorphism and diabetic nephropathy.. J Nephropathol..

[3] Rouhi H, Ganji F (2013). Effects of N-acetyl cysteine on serum lipoprotein (a) and proteinuria in type 2 diabetic patients.. J Nephropathol..

[4] Tavafi M (2013). Complexity of diabetic nephropathy pathogenesis and design of investigations.. J Renal Inj Prev..

[5] Nasri H, Nematbakhsh M, Ghobadi S, Ansari R, Shahinfard N, Rafieian-Kopaei M (2013). Preventive and curative effects of ginger extract against histopathologic changes of gentamicin-induced tubular toxicity in rats.. Int J Prev Med..

[6] Tzeng TF, Liou SS, Chang CJ, Liu IM (2013). The Ethanol Extract of Zingiber zerumbet Attenuates Streptozotocin-Induced Diabetic Nephropathy in Rats.. Evid Based Complement Alternat Med..

[7] Hajivandi A, Amiri M (2014). World Kidney Day 2014: Kidney disease and elderly. J Parathyr Dis.

[8] Tavafi M (2013). Diabetic nephropathy and antioxidants.. J Nephropathol..

[9] Nasri H (2013). Acute kidney injury and beyond.. J Renal Inj Prev..

[10] Rafieian-Kopaie M, Baradaran A (2013). Teucrium polium and kidney.. J Renal Inj Prev..

[11] Morales AI, Detaille D, Prieto M, Puente A, Briones E, Arevalo M (2010). Metformin prevents experimental gentamicin-induced nephropathy by a mitochondria-dependent pathway.. Kidney Int..

[12] Amini FG, Rafieian-Kopaei M, Nematbakhsh M, Baradaran A, Nasri H (2012). Ameliorative effects of metformin on renal histologic and biochemical alterations of gentamicin-induced renal toxicity in Wistar rats.. J Res Med Sci..

[13] Rafieian-Kopaei M, Baradaran A (2013). Combination of metformin with other antioxidants may increase its renoprotective efficacy.. J Renal Inj Prev..

[14] Baradaran A, Rafieian-kopaei M (2013). Histopathological study of the combination of metformin and garlic juice for the attenuation of gentamicin renal toxicity in rats.. J Renal Inj Prev..

[15] Kim J, Shon E, Kim CS, Kim JS (2012). Renal podocyte injury in a rat model of type 2 diabetes is prevented by metformin.. Exp Diabetes Res..

[16] Kari J (2012). Epidemiology of chronic kidney disease in children.. J Nephropathol..

[17] Rafieian-Kopaei M, Nasri H (2013). Ginger and diabetic nephropathy.. J Renal Inj Prev..

[18] Rafieian-Kopaei M, Baradaran A (2003). Teucrium polium and kidney.. J Renal Inj Prev..

[19] Behradmanesh S, Rafieian-kopaei M (2006). Effect of Salvia officinalis on diabetic patients.. J Renal Inj Prev..

